# Cardiac mesenchymal progenitors differentiate into adipocytes via *Klf4* and *c-Myc*

**DOI:** 10.1038/cddis.2016.31

**Published:** 2016-04-14

**Authors:** D Kami, T Kitani, T Kawasaki, S Gojo

**Affiliations:** 1 Department of Regenerative Medicine, Kyoto Prefectural University of Medicine, Kyoto, Japan; 2 Department of Cardiovascular Medicine, Graduate School of Medical Science, Kyoto Prefectural University of Medicine, Kyoto, Japan

## Abstract

Direct reprogramming of differentiated cells to pluripotent stem cells has great potential to improve our understanding of developmental biology and disorders such as cancers, and has implications for regenerative medicine. In general, the effects of transcription factors (TFs) that are transduced into cells can be influenced by pre-existing transcriptional networks and epigenetic modifications. However, previous work has identified four key TFs, *Oct4, Sox2, Klf4* and *c-Myc*, which can reprogram various differentiated cells to generate induced pluripotent stem cells. Here, we show that in the heart, the transduction of cardiac mesenchymal progenitors (CMPs) with Klf4 and c-Myc (KM) was sufficient to drive the differentiation of these cells into adipocytes without the use of adipogenic stimulation cocktail, that is, insulin, 3-isobutyl-1-methylxanthine (IBMX) and dexamethasone. KM-transduced CMPs exhibited a gradually increased expression of adipogenic-related genes, such as *C/Ebpα*, *Pparγ* and *Fabp4*, activation of the peroxisome proliferator-activated receptor (PPAR) signaling pathway, inactivation of the cell cycle-related pathway and formation of cytoplasmic lipid droplets within 10 days. In contrast, NIH3T3 fibroblasts, 3T3-L1 preadipocytes, and bone marrow-derived mesenchymal stem cells transduced with KM did not differentiate into adipocytes. Both *in vitro* and *in vivo* cardiac ischemia reperfusion injury models demonstrated that the expression of KM genes sharply increased following a reperfusion insult. These results suggest that ectopic adipose tissue formation in the heart following myocardial infarction results from CMPs that express KM following a stress response.

Adipocyte differentiation, that is, adipogenesis, has been extensively investigated, and its regulation via transcriptional cascades has been described for *in vitro* model systems.^1^ The adipogenic transcriptional cascade consists of two waves. The first wave converges at the CCAAT/enhancer-binding protein (C/Ebp)*β*/*γ*, which induces the second wave consisting of nuclear receptor peroxisome proliferator-activated receptor (Ppar)*γ* and C/EBP*α* activity. In addition, *c-Myc* is periodically expressed during the early phase of adipogenesis.^2^ Krüppel-like factor (*Klf*) family members include both repressors and activators of adipogenesis, and are activated during the first wave.^3^ KLF4 and c-MYC (KM) coordinately bind the promoters of genes that are activated during the reprogramming of differentiated cells to pluripotency.^4^ Whether KM work together in adipogenesis has not been examined.

Mesenchymal stem cells (MSCs) are multipotent cells with a capacity to differentiate to mesodermal lineages and show a vigorous proliferation capacity under conventional culture conditions.^5^ The criteria for identifying MSCs include adherence to a plastic dish, a characteristic surface profile and differentiation capacity *in vitro*.^6^ Although most prior reports have identified bone marrow as the origin for MSCs, other organs including adipose tissue^7^ and the heart^8, 9^ also harbor fibroblasts that fulfill the criteria for MSCs. MSCs derived from different organs demonstrate varying capacities for proliferation and differentiation.^10^ Although several reports have demonstrated adipose tissue formation in the myocardium following reperfusion therapy for ischemic heart diseases,^11, 12, 13, 14^ it is unclear how fat depositions in the heart are generated.

Direct reprogramming of differentiated cells using specific transcription factors (TFs) opens the door to understanding the mechanisms underlying development and the pathogenesis of various disorders, and has applications in regenerative medicine.^15, 16^ Transdifferentiation or direct conversion, which occurs when a differentiated cell type is reprogrammed to another cell type, could be implemented via the same strategy of using a set of TFs to generate cardiomyocytes, neurons and so on.^17, 18^ Moreover, there are similarities and overlaps between the pathways for the generation of induced pluripotent stem cells (iPSCs) and tumorigenesis, such as a mesenchymal-to-epithelial transition.^19^ Recently, it was reported that partial reprogramming of differentiated cells using four reprogramming TFs (*Oct4, Sox2, Klf4* and *c-Myc* (OSKM)) *in vivo* could generate tumors via epigenetic reprogramming.^20^ Direct reprogramming can shed light on cancer biology, and vice versa.

Transcriptional cascades definitively determine cell fate during reprogramming to pluripotency and normal differentiation. We examined whether murine cardiac mesenchymal progenitors (CMPs)^9^ expressing Sca-1 antigen and TFs associated with cardiomyocytes can differentiate into adipocytes and how the process is regulated. Elucidating the mechanisms underlying adipose tissue generation in the heart should help us to understand the pathophysiologies of ischemic reperfusion injury (IRI) myocardial infarction (MI).

## Results

### Transduction of OSKM into CMPs is sufficient to induce their differentiation into adipocytes

**Figure 1:**
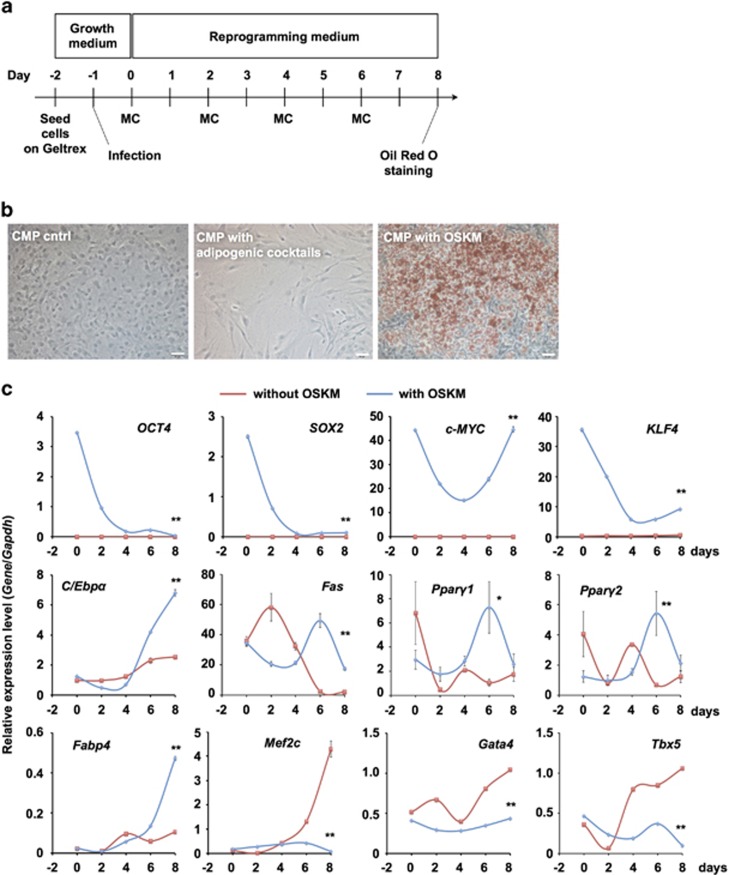
OSKM-transduced CMPs differentiated into adipocytes. (**a**) Schematic representation of the adipocyte differentiation method. MC, medium change. Growth medium indicates the basal medium for each cell line, and reprogramming medium indicates KO-DMEM-based medium. (**b**) Phase contrast microscope images. CMPs treated with OSKM Sendai virus (CMP with OSKM) clearly accumulated large cytosolic lipid droplets at day 8. These droplets were stained with Oil Red O. Untreated CMPs (CMP cntrl) and those treated with adipogenic stimulation cocktails (CMP with adipogenic cocktails) did not form cytosolic lipid droplets at day 8 and were not stained with Oil Red O. The white bar indicates 50 *μ*m. (**c**) qRT-PCR analysis of the expression of each gene in CMPs on each day. Individual RNA expression levels were normalized to *Gapdh* expression. Error bars indicate S.E. (*n*=3). * and ** indicate significant changes compared with untreated controls at day 8 (*P*<0.05 and 0.01, respectively)

To test our hypothesis that reprogrammed CMPs can differentiate into other cell types, we transduced CMPs with Sendai virus encoding OSKM. We modified standard reprogramming medium by removing leukemia inhibitory factor to avoid the generation of iPSCs, following a previous report (Figure 1a).^21^ Nine days after infection (day 8), OSKM-transduced CMPs (OSKM-CMPs) formed cytoplasmic lipid droplets, which were not formed by untreated CMPs (CMP control) or CMPs treated with adipogenic differentiation cocktails (CMP with adipogenic cocktails) (Figure 1b). The lipid droplets in OSKM-CMPs were clearly stained by Oil Red O (Figure 1b). Next, to identify gene expression in reprogrammed CMPs, we performed quantitative reverse transcription polymerase chain reaction (qRT-PCR) analysis (Figure 1c). The expression levels of *Oct4* and *Sox2* in OSKM-CMPs decreased at day 2 and were maintained at a low level thereafter. *Klf4* and *c-Myc* expression in OSKM-CMPs also decreased at day 2. The expression levels of the adipogenic-related genes *C/Ebpα* and *Fabp4* in OSKM-CMPs increased at day 4. The expression levels of Fas, Ppar*γ*1 and Ppar*γ*2 in OSKM-CMPs were higher than those in untreated CMPs at day 6. Moreover, the expression levels of the cardiac-related genes *Mef2c*, *Gata4* and *Tbx5* in CMP controls increased, but these genes were not expressed in OSKM-CMPs.

### Transduction of OSKM into NIH3T3 fibroblasts is insufficient to induce their differentiation into adipocytes

**Figure 2:**
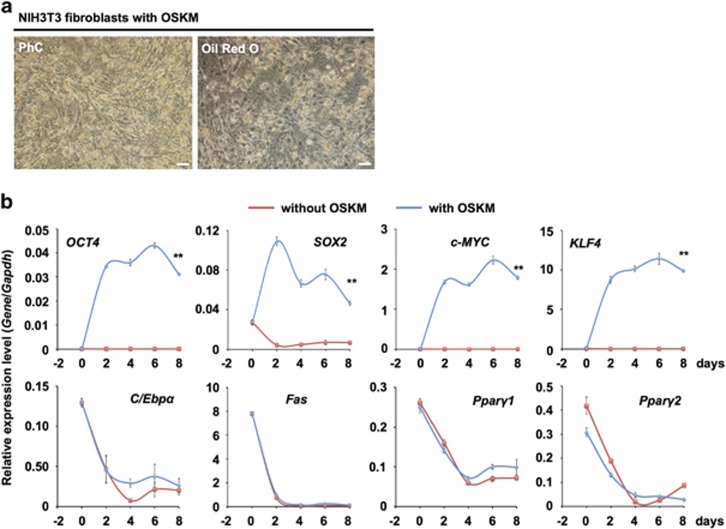
OSKM-transduced NIH3T3 fibroblasts did not differentiate into adipocytes. (**a**) Phase contrast microscope images. NIH3T3 fibroblasts treated with OSKM Sendai virus (NIH3T3 fibroblasts with OSKM) did not form cytosolic lipid droplets at day 8 and were not stained with Oil Red O. White bar indicates 50 *μ*m. PhC, phase contrast. (**b**) qRT-PCR analysis of the expression of each gene in NIH3T3 fibroblasts on each day. Individual RNA expression levels were normalized to *Gapdh* expression. Error bars indicate S.E. (*n*=3). * and ** indicate significant changes compared with untreated controls at day 8 (*P*<0.05 and 0.01, respectively)

Next, we transduced OSKM into NIH3T3 fibroblasts. At day 8, NIH3T3 fibroblasts changed in shape from fibroblast-like cells to round cells; however, there were no iPSC-like colonies or Oil Red O-positive cells (Figure 2a). Expression of OSKM genes at day 2 increased rapidly; however, the expression levels of the adipogenic genes *Fas*, *C/Ebpα* and *Pparγ2* decreased steeply from day 2 (Figure 2b). In particular, *C/Ebpα* and *Fas* expression did not differ from that of the control (without OKSM). These results showed that OSKM-transduced NIH3T3 fibroblasts did not differentiate into adipocytes.

### Microarray analysis of OSKM-CMPs

**Figure 3:**
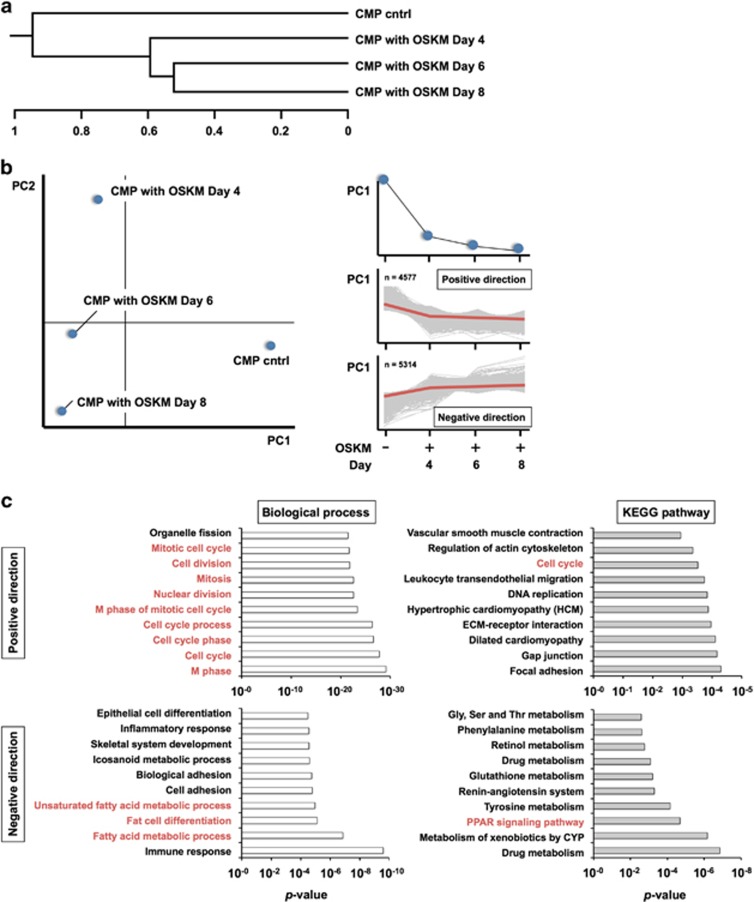
Global gene expression of OSKM-transduced CMPs. (**a**) Hierarchical clustering analysis of OSKM-transduced CMPs on each day by NIA array analysis. CMP cntrl indicates untreated CMPs. (**b**) PCA by NIA array analysis. CMPs are categorized based on the principal component 1 (PC1) direction (left). A total of 4577 probes were in the positive direction on PC1, indicating decreased expression over time, and 5314 probes were in the negative direction of PC1, indicating increased expression over time. (**c**) Genes in the PC1-positive and -negative directions were categorized based on biological processes using GO annotations (white bars) and KEGG pathways (gray bars)

To analyze global gene expression in OSKM-CMPs, we performed microarray analysis using an Agilent mouse microarray chip and the NIA Array Analysis website.^22^ Based on hierarchical clustering analysis of gene expression, OSKM-CMPs could be clearly discriminated from CMP controls (Figure 3a). In addition, principal component analysis (PCA) of gene expression showed that the OSKM-CMPs were different from the CMP controls and gradually shifted from right to left on the PC1 axis in a time-dependent manner (Figure 3b). Furthermore, a group of genes with decreasing expression over time (positive direction along PC1, 4577 probes) and a group with increasing expression over time (negative direction along PC1, 5314 probes) were observed (Figure 3b). These genes were categorized based on gene ontology (GO) annotations and Kyoto Encyclopedia of Genes and Genomes (KEGG) pathways (Figure 3c). Many genes showing decreasing expression over time (PC1-positive direction) were assigned to functional categories related to cell cycling and cell division. In addition, many of the genes showing decreasing expression over time were assigned to functions related to focal adhesion and regulation of the actin cytoskeleton. Otherwise, the genes showing increasing expression over time (PC1-negative direction) were functionally related to adipocyte differentiation, including saturated and unsaturated fatty acid metabolism, fat cell differentiation and the PPAR signaling pathway. These results strongly indicated that OSKM-CMPs differentiated into adipocytes.

### *Klf4* and *c-Myc* have important roles in the differentiation of CMPs into adipocytes

**Figure 4:**
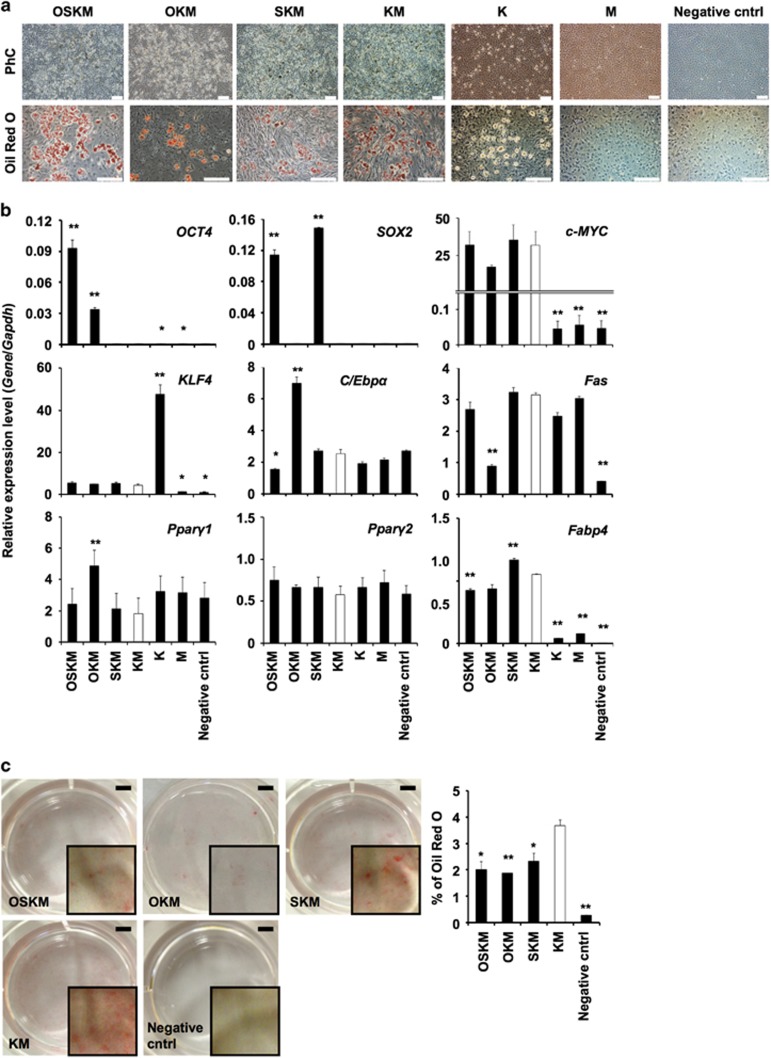
Adipocyte differentiation properties of TF-transduced CMPs. (**a**) Phase contrast microscope images. CMPs were transduced using a combination of OSKM, SKM, KM, K and M Sendai virus. The white bar indicates 200 *μ*m. (**b**) qRT-PCR analysis of the expression of each gene in CMPs at day 8. Individual RNA expression levels were normalized to *Gapdh* expression. Error bars indicate S.E. (*n*=3). * and ** indicate significant changes compared with KM-treated CMPs (white box, *P*<0.05 and 0.01, respectively). (**c**) Calculation of Oil Red O staining area. Each well image was captured using a Keyence BZ-X700 digital microscope. The black bar indicates 5 mm (left). The graph shows the percentages of the total area that were positive for Oil Red O staining (right). Error bars indicate S.E.; * and ** indicate significant changes (*P*<0.05 and 0.01, respectively). OKM: *Oct4, Klf4* and *c-Myc*; SKM: *Sox2*, *Klf4* and *c-Myc*; KM: *Klf4* and *c-Myc*; K: *Klf4*; M: *c-Myc*. Negative cntrl: untreated CMPs

To determine which of the reprogramming factors among OSKM were critical for CMP differentiation into adipocytes, we examined the effect of removing each factor. We searched genetic databases for information regarding gene expression during adipogenesis in 3T3-L1 cells. The available information from previous studies indicated that *Klf4* was expressed before adipogenic stimulation and that *c-Myc* sharply rose up in response, then the expression of both genes decreased to become undetectable at 1 week, at which time lipid-laden adipocytes were macroscopically recognized using the standard protocol (GSE34150). Both *Klf4* and *c-Myc* have been reported to be adipocyte differentiation-related factors.^3, 23^ We withdrew OSKM sequentially. Neither the withdrawal of *Oct4* nor that of *Sox2* influenced adipogenic differentiation based on Oil Red O staining. The withdrawal of either *Klf4* or *c-Myc* decreased the percentage of Oil Red O-positive cells 9 days after infection, compared with that observed when these factors were present (Figure 4a). The expression levels of genes related to adipogenesis including *C/Ebpα*, *Fas* and *Pparγ2* in TF(s)-transduced CMPs at day 8 showed a similar pattern, regardless of the TFs used except OKM-infected CMP. In OKM-infected CMP cells, the expression of both C/Ebp*α* and Ppar*γ*1 was upregulated, but Fas, which is involved in lipogenesis,^24^ was downregulated. C/Ebpα is mainly attributed to a role in insulin sensitivity in adipocyte differentiation,^25^ and Ppar*γ*2, but not Ppar*γ*1, has an essential role in adipogenic differentiation *in vitro.*^26^ Overexpression of c-MYC elicits p53-dependent apoptosis in primary fibroblasts.^27, 28^ Infection of CMP cells might result in apoptosis, therefore the expression level of c-MYC was almost the same as that of the negative control. KLF4 might be required to suppress p53 and c-Myc-induced apoptosis. *Fabp4*, which is expressed during terminal differentiation,^29^ was not expressed in K- and M-transduced CMPs (Figure 4b). This observation might be ascribed to the inability of those CMPs to differentiate into mature lipid-laden adipocytes. Furthermore, KM genes in CMPs resulted in a greater relative area of Oil Red O-positive cells than that observed for the transduction of OSKM, OKM and SKM (Figure 4c). These results showed that the combination of *Klf4* and *c-Myc* is indispensable for the differentiation of CMPs into adipocytes.

### *Klf4* and *c-Myc* do not induce adipogenic differentiation of MSCs

**Figure 5:**
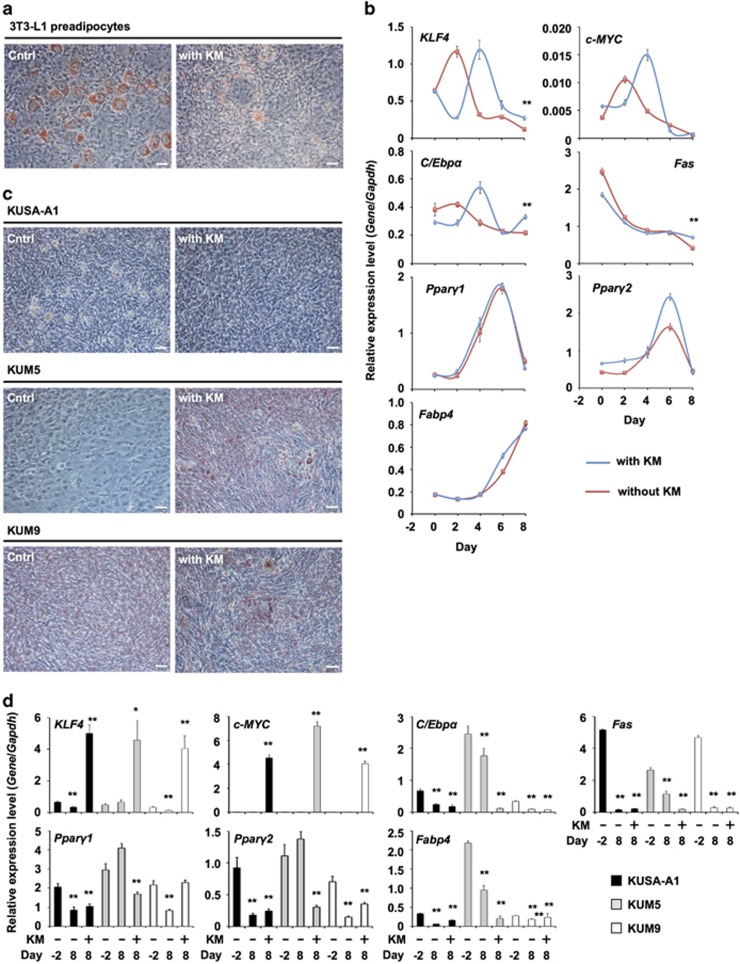
Adipocyte differentiation properties of KM-transduced 3T3-L1 preadipocytes and MSCs derived from bone marrow (KUSA-A1, KUM5 and KUM9 cells). (**a**) Phase contrast microscope images. 3T3-L1 preadipocytes were transduced with KM Sendai virus. One day after infection, cells were cultured in reprogramming medium for 8 days. At day 8, cells were fixed and stained with Oil Red O. The white bar indicates 50 *μ*m. Cntrl indicates untreated 3T3-L1 preadipocytes. (**b**) qRT-PCR analysis of the expression of each gene in 3T3-L1 preadipocytes on each day. Individual RNA expression levels were normalized to *Gapdh* expression. Error bars indicate S.E. (*n*=3). * and ** indicate significant changes compared with KM-treated 3T3-L1 cells at day 8 (*P*<0.05 and 0.01, respectively). (**c**) Phase contrast microscope images. MSCs derived from mouse bone marrow were transduced with KM Sendai virus. One day after infection, cells were cultured in reprogramming medium for 8 days. At day 8, cells were fixed and stained with Oil Red O. The white bar indicates 50 *μ*m. Cntrl indicates untreated MSCs. (**d**) qRT-PCR analysis of the expression of each gene in MSCs KUSA-A1 (black bars), KUM5 (gray bars) and KUM9 (white bars) at day 8. Individual RNA expression levels were normalized to *Gapdh* expression. Error bars indicate S.E. (*n*=3). * and ** indicate significant changes from untreated control cells (KM–, day –2) (*P*<0.05 and 0.01, respectively)

To test the ability of other cell types to differentiate into adipocytes via KM transduction, we used the same methods to induce adipocyte differentiation (Figure 1a). KM genes did not increase the frequency of adipocyte differentiation in 3T3-L1 preadipocytes (Figure 5a). All of the adipogenic genes exhibited a similar expression pattern in both non-treated and KM-transduced 3T3-L1 preadipocytes (Figure 5b). Furthermore, we examined whether other mouse multipotent MSCs derived from bone marrow (KUSA-A1, KUM5 and KUM9 cells) could be induced to undergo differentiation to adipocytes by KM transduction. Cells treated with KM did not show any formation of lipid droplets in the cytosol (Figure 5c). The cells exhibited high expression levels of *c-Myc* and *Klf4*, and low or unchanged expression levels of adipogenic genes such as *C/Ebpα*, *Fas*, *Pparγ2* and *Fabp4* at day 8 (Figure 5d). Interestingly, the expression of all adipogenic genes in these MSCs decreased at day 8. These results showed that these cells were not able to differentiate into adipocytes via KM. The expression levels of a set of TFs in bone marrow-derived mesenchymal cell lines, including KUSA-A1, KUM5 and KUM9 cells, were determined relative to the expression of *Gapdh* from cardiac tissues. All TFs related to adipogenesis did not increase following exogenous KM gene transfer, suggesting that the induction of adipogenesis by KM gene transfer could be specific to CMPs.

### *Klf4* and *c-Myc* were induced by ischemia reperfusion injury *in vitro* and *in vivo*

**Figure 6:**
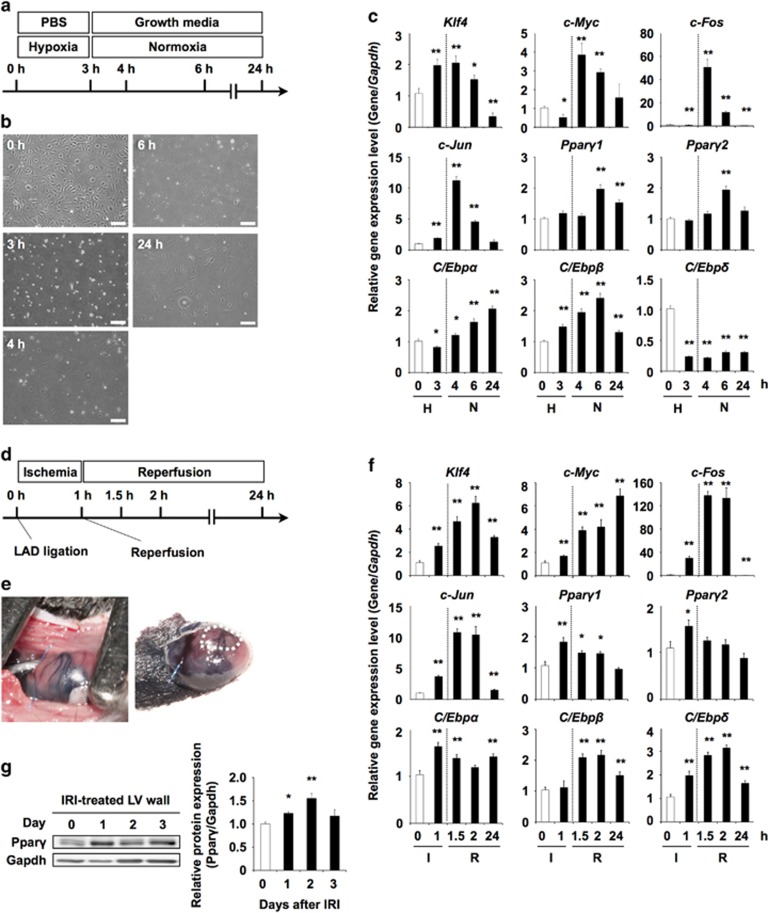
*In vitro* and *in vivo* IRI model. (**a**) Schematic representation of the *in vitro* IRI method. PBS, phosphate-buffered saline. Growth medium indicates the basal medium for CMP. Hypoxia indicates 1% O_2_, 5% CO_2_, balance N_2_ conditions in a hypoxic chamber, and normoxia indicates 21% O_2_, 5% CO_2_ conditions. (**b**) Phase contrast microscope images of CMPs under hypoxic conditions. (**c**) qRT-PCR analysis of the expression of each gene in CMPs. Error bars indicate S.E. (*n*=3). * and ** indicate significant changes compared with CMPs at 0 h (white box, *P*<0.05 and 0.01, respectively). H indicates hypoxia condition; N indicates hormoxia condition. (**d**) Schematic representation of the *in vivo* IRI method. (**e**) Photograph of open-chest mouse with 8-0 Prolene suture thread on the LAD. (**f**) qRT-PCR analysis of the expression of each gene in an IRI model LV heart. Error bars indicate S.E. (*n*=3). * and ** indicate significance (*P*<0.05 and 0.01, respectively). ‘I' indicates ischemic condition; ‘R' indicates reperfusion condition. (**g**) Western blotting of IRI model LV heart. Error bars indicate S.E. (*n*=3). * and ** indicates statistically significant differences at 0 h (white box, *P*<0.05 and 0.01, in the order described)

CMPs were exposed to IRI model culture conditions *in vitro* (Figure 6a). CMPs immediately detached from the tissue culture dish under hypoxic conditions, and detached CMPs re-attached to the culture dish under normoxic conditions (Figure 6b). CMPs expressed the hypoxia-induced gene *Hif1α* at 3 h, and expression returned to baseline levels at 24 h (Supplementary Figure 1), indicating that the culture system could successfully mimic IRI. The expression level of *Klf4* increased at 3 h after hypoxia, remained high until 6 h under normoxia, then returned to the baseline level, whereas the expression of *c-Myc* increased sharply just after normoxic conditions were applied. HIF1, the abundance of which indicates the extent of the ischemic insult, is upregulated by the inhibition of proteolysis in cardiac IRI,^30^ and its abundance can be measured by western blotting. To assess the link between a set of TFs and IRI using a consistent methodological approach, qRT-PCR was chosen. As a surrogate marker for IRI, instead of *Hif-1*, the gene expression levels of *c-Fos* and *c-Jun*, the protein products of which form the TF AP1, were examined. The expression of both *c-Fos* and *c-Jun* drastically and temporarily increased just after exposure to normoxic conditions.

Moreover, we determined that KM was involved in *in vivo* murine cardiac IRI (Figures 6d–f). Injured murine ventricles acutely and temporarily expressed *Klf4* and *c-Myc* in IRI, similar to the pattern observed for *in vitro* IRI. The expression levels of both *c-Fos* and *c-Jun* were transiently increased 1 h after ischemia, and increased after reperfusion, indicating that *Klf4* might be involved in the cellular response following the insult as early as *c-Fos* and *c-Jun*. Platelet-derived growth factor, which is involved in cardiac IRI, induces *c-Myc* expression via an AP1-dependent signaling pathway under *in vitro* culture conditions.^31^ The kinetics of *c-Fos* and *c-Jun* expression showed an earlier response to IRI than was found for *c-Myc*, suggesting that AP1, which is composed of c-Fos and c-Jun, might be an upstream regulator of *c-Myc*. The expression of *C/Ebpβ* and *C/Ebpδ* gradually increased; however, at 24 h the gene expression levels of both *C/Ebpβ* and *C/Ebpδ* returned to baseline, whereas *C/Ebpα* gene expression was increased and maintained at a higher level 3 h after left anterior descending artery (LAD) ligation. The expression levels of *Pparγ1* and *Pparγ2* transiently increased at 1 h (Figure 6f), and the expression of PPAR*γ* protein transiently increased at days 1 and 2 (Figure 6g). However, reperfused hearts were not stained by Oil Red O staining (Supplementary Figure 2).

### Regional expression of adipogenic-related genes in a RA, an AAR and the AON

**Figure 7:**
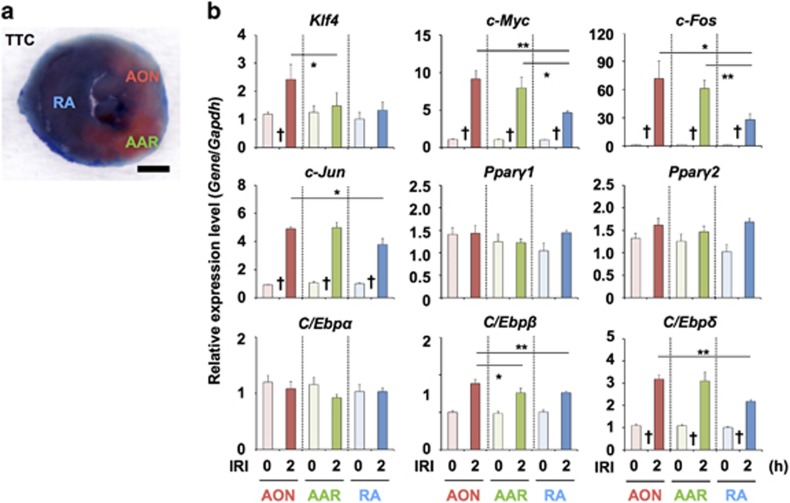
Gene expression in tested areas of ischemia reperfusion injury hearts. (**a**) TTC staining and Oil red O staining of ischemic reperfusion heart. RA, AOR and AAR indicate RA, AON and AAR, respectively. Black bar: 1 mm. (**b**) qRT-PCR analysis for the expression of genes in the ischemic reperfusion mouse heart on each day. Individual RNA expression levels were normalized to the respective mouse *Gapdh* expression levels. Error bars indicate S.E. (*n*=3). * and ** indicates statistically significant differences in each color box. (*P*<0.05 and 0.01, in the order described). † also indicates statistically significant difference from IRI non-treatment heart (0 h) (*P*<0.05 and 0.01, in the order described). Relative gene expression in the RA at 0 h is regarded as 1

To evaluate RNA expression in the LV wall of the IRI model in detail at 2 h after LAD ligation, a set of TFs related to adipogenesis was examined by qRT-PCR for each area consisting of the area of necrosis (AON), an area at risk (AAR) and a remote area (RA), which were defined by double staining with 2,3,5-triphenyltetrazolium chloride (TTC) and Evans blue (Figure 7a). *Klf4* expression significantly increased in the AON, and *c-Myc* expression significantly increased in all areas over time. The first wave of TFs (C/Ebp*β* and C/Ebp*δ*) for adipogenesis was significantly raised in all areas. In contrast, expression of the second wave of TFs (*Pparγ2*, and *C/Ebpα*) did not differ in AON and AAR compared with that in RA. Comparison of gene expression among areas revealed that *Klf4* expression in AON was significantly higher than that in RA, and *c-Fos*, *c-Jun,* and *c-Myc* expression levels in AON and AAR were significantly higher than those in RA. The gene expression of the first wave of TFs in AON was significantly higher than that in RA, and the gene expression of the second wave of TFs was not significantly different among the three areas. The surrogate TFs (*c-Fos* and *c-Jun*) showed significantly increased expression in AON and AAR compared with that in RA, which was the same tendency as that observed for *Klf4*, *c-Myc*, *C/Ebpβ* and *C/Ebpδ*, suggesting that adipogenesis might be initiated via KM induction in this cardiac IRI model (Figure 7b).

## Discussion

We demonstrated that CMPs could effectively differentiate into adipocytes via two TFs, *Klf4* and *c-Myc*, without adipogenic stimulation. Neither *Klf4* nor *c-Myc* transduction alone resulted in CMPs differentiating into adipocytes. These results suggested that KM proteins in CMPs coordinately regulate adipogenesis. Interestingly, this new protocol was only effective in CMPs, but not in MSCs from bone marrow and 3T3-L1 preadipocytes. CMPs did not differentiate into adipocytes when treated with adipogenic stimulation cocktails, contradicting the hypothesis that CMPs contain adipogenic progenitors or stem cells. These results might indicate that the route to adipocyte differentiation is not uniform as defined by the *in vitro* cellular model, but rather that it depends upon the cell type and environment. The increased expression levels of both *Klf4* and *c-Myc* in IRI models might be due to intracardiac fatty degeneration following MI.

Previous *in vitro* studies of the molecular pathways underlying adipogenesis are based on limited adipogenic cell lines.^32, 33, 34, 35^ Adipogenesis involves two distinct waves of TF expression and six defined differentiation stages: mesenchymal precursors, committed preadipocytes, growth-arrested preadipocytes, mitotic clonal expansion, terminal differentiation and mature adipocytes.^36, 37, 38^ Preadipocytes differentiate into lipid-laden and insulin-sensitive adipocytes upon the addition of exogenous adipogenic stimulation cocktails in confluent culture growth conditions. *Klf4* is one of the earliest TFs in the first wave and is regulated by cAMP^3^ and the JAK-STAT pathway, mechanisms that maintain the pluripotency of embryonic stem cells.^39^ As the Jak-Stat pathway is activated by external stimuli such as reactive oxygen species (ROS),^40^ *Klf4* mediates the response to external stress.^41^ KLF4 directly transactivates the *C/EBPβ* gene by binding to the promoter region, and is a key TF in the first wave that relays the signal to PPAR*γ*, a central factor in adipogenesis.^3^ However, within 1 h of adipogenic stimulation of confluent 3T3-L1 cells, *c-Myc* is rapidly and highly expressed, along with *c-Fos* and *c-Jun*.^23^ Constitutively overexpressed *c-Myc* inhibits the differentiation of 3T3-L1 cells, possibly by precluding the entry of cells to a distinct predifferentiation stage in G_0_/G_1_.^42^ The peak in the expression of *c-Myc* might function as an amplifier of the expression of other genes to surpass the threshold from a stable, low-level position in adipocytes and not as an activator of the cell cycle.^43^ In this experiment, neither *Klf4* nor *c-Myc* transduction alone induced adipogenesis from CMPs, suggesting that KM cooperatively function to induce adipogenesis.

Little is known about *in vivo* adipogenesis or *de novo* adipocyte generation, which is referred to as hyperplasia in terms of tissue growth, owing to the post-mitotic nature of mature adipocytes. In adipose tissue, resident MSCs are considered to be a major source for adipocyte generation.^36^ Some studies have reported *in vivo* adipocyte differentiation from MSCs, which expressed similar cell surface antigens to those expressed by the CMPs in this study.^44, 45^ Recently, myocardium-derived stem/progenitor cells such as cardiac stem cells and CMPs have been reported by several institutes.^9, 46, 47, 48^ CMPs, which we isolated from murine hearts and defined as a Sca-1-positive population, expressed a similar surface antigen profile to that of MSCs,^6^ except for CD73 and CD34. The origin of each MSC influences its molecular phenotype, including the transcriptional network, epigenetic landscape and subsequent differentiation potential.^10, 38^ CMPs are a distinct population from bone marrow, adipose tissue or skin-derived MSCs, with an expression profile of TFs characteristic of the heart. Only KM induced the differentiation of CMPs into adipocytes, potentially owing to the default settings of the TF network.

Baroldi *et al.*^11^ reported adipose tissue formation in the excised heart during transplant surgery, and this was termed lipomatous metaplasia. Another study using the recipient heart in transplantation showed consistent ectopic fat formation, representing 84% of healed MI.^12^ Imaging analyses in patients with a history of MI using either computed tomography^13^ or magnetic resonance imaging^14^ have demonstrated a similar prevalence of ectopic fat formation, which was found in approximately 65% of individuals. These reports suggest that adipose formation in the myocardium should be a more common pathology than is currently recognized. A new mechanism of arrhythmogenesis in ventricular tachycardia proposes that intramyocardial adipose tissue hinders myocardial conduction and worsens local electrophysiological properties, which in turn results in an increased propensity for ventricular tachycardia.^49^ The KM genes transduced into CMP maintained high expression for about 1 week, resulting in differentiation of the CMPs to lipid-laden adipocytes and activation of the second wave of TFs for adipogenesis. In contrast, cardiac IRI temporarily induced *Klf4* and *c-Myc* expression, which sharply fell and disappeared after only a few days, resulting in failure to maintain the second wave and generate adipocytes. During left ventricular (LV) remodeling post MI, the renin–angiotensin–aldosterone system (RAAS) is activated, which leads to AP1 activation^50^ and might result in *c-Myc* induction.^51^ Angiotensin II can induce *Klf4* expression in cardiac fibroblasts including CMPs.^52^ Although RAAS does not induce a high expression of KM such as that which we observed during *in vitro* adipogenesis in this study, the low level of KM expression over a long period induced by RAAS might slowly form adipogenic enhanceosomes at enhancer regions for late-acting TFs in adipogenesis, such as *Pparγ.*^37^ Inhibition of either *Klf4* or *c-Myc* induction might be a novel strategy to treat LV remodeling post MI.

Global mRNA profiling of the myocardium after IRI has revealed that *Klf* family members, including *Klf4* and *c-Myc*, exhibit significantly increased expression following ischemia and additional increases after reperfusion.^53^ Ischemic events generate interleukin 6 in the heart, activating STAT3,^54^ which is linked to *Klf4* expression.^39^ However, ROS, which have been characterized as negative factors in reperfusion injuries, are involved in signal transduction in many biological processes, including inflammation, stemness and differentiation, cancer, and aging.^55^ The thioredoxin family member nucleoredoxin (NRX), which is a redox sensor regulated by ROS, interacts with dishevelled (Dvl) under a reduction state. The oxidized form of NRX liberates Dvl, which in turn stabilizes *β*-catenin, leading to the transcription of WNT target genes including *c-Myc*.^56^ Consistent with the aforementioned studies on signal transduction, myocardial ischemia led to increased expression of *Klf4* and reperfusion stimulated *c-Myc* expression. These results strongly suggest that the two TFs KLF4 and c-MYC in CMPs are causative factors for intracardiac adipogenesis following myocardial reperfusion. The regional assessment revealed that the expression levels of *Klf4*, *c-Myc*, *c-Fos* and C/Ebpδ in AON and AAR were raised more than those in RA, suggesting that the adipogenic differentiation process had already been launched in the area directly affected by the insult of ischemia and reperfusion at 2 h.

MSCs can be isolated from various tissue types including bone marrow, adipose tissue, heart and skeletal muscle. However, the characteristics and epigenetic background of these MSCs differ.^10^ Transduction of CMPs with KM genes was highly effective in inducing their differentiation into adipocytes, whereas transducing the same genes into MSCs derived from other tissues did not induce them to differentiate. Furthermore, these phenomena might provide a basis for ectopic fat formation in ischemic hearts. Understanding CMP adipogenesis should shed light on post-MI and IRI pathophysiology and facilitate the development of better treatments for these disorders.

## Materials and Methods

### Materials

Geltrex and basic fibroblast growth factor (bFGF) were purchased from Life Technologies (Carlsbad, CA, USA). The CytoTune-iPS ver. 1.0 Sendai Reprogramming Kit was purchased from DNAVEC (Ibaraki, Japan). Oil Red O powder was purchased from Nacalai Tesque, Inc. (Kyoto, Japan). Percoll Plus was purchased from GE Healthcare UK (Buckinghamshire, England). The adipogenic stimulation cocktail ingredients insulin, IBMX, and dexamethasone were purchased from Sigma-Aldrich (St. Louis, MO, USA).

### Cell preparation

Experimental procedures and protocols were approved by the Animal Experiment Ethics Committee of the Kyoto Prefectural University of Medicine. Murine CMPs were isolated from wild-type C57BL/6 mouse hearts (10- to 16-week-old).^9^ Briefly, the mice were killed by deep anesthesia with pentobarbital. The hearts were excised, and atria were used in this study. The minced tissue fragments were digested twice for 30 min at 37 °C with 0.2% (w/v) type II collagenase and 0.01% (w/v) DNase I (Worthington Biochemical, Lakewood, NJ, USA). After digestion, cells were passed through a 70-*μ*m filter to remove debris and transferred to Dulbecco's modified Eagle's medium (DMEM)/F12 supplemented with 10% (v/v) fetal bovine serum (FBS) (Life Technologies). The cells were collected and size fractionated on a 30–70% Percoll gradient to obtain CMPs expressing the Sca-1 antigen. CMPs were seeded on 60-mm collagen I-coated dishes (Asahi Glass, Tokyo, Japan) in DMEM/F12 supplemented with 10% (v/v) FBS and 20 ng/ml bFGF. The medium was changed every 3 days.

### Cell culture and adipocyte differentiation

CMPs were cultured in DMEM/F12 supplemented with 10% (v/v) FBS and 20 ng/ml bFGF in a humidified atmosphere containing 5% CO_2_. NIH3T3 fibroblasts and MSCs derived from bone marrow KUM5, KUM9 and KUSA-A1^33^ were cultured in DMEM (Wako Chemical Co., Osaka, Japan) supplemented with 10% (v/v) FBS in a humidified atmosphere containing 5% CO_2_. The 3T3-L1 preadipocytes were cultured in minimum essential media (MEM) (Life Technologies) supplemented with 10% (v/v) FBS in a humidified atmosphere containing 5% CO_2_. For adipocyte differentiation, before viral transduction, cells were seeded at 0.5 × 10^5^ per well on Geltrex-coated six-well plates (1:40, Life Technologies) in growth medium (day –2). On the next day (day –1), cells were transduced using the CytoTune-iPS ver. 1.0 Sendai Reprogramming Kit according to the manufacturer's recommendations. At 24 h after transduction (day 0), cells were transferred to reprogramming media, that is, knockout DMEM (KO-DMEM) with 5% (v/v) knockout serum replacement, 15% (v/v) FBS, 1% (v/v) GlutaMAX solution, 1% (v/v) nonessential amino acids solution and 0.1 mM *β*-mercaptoethanol (all components obtained from Life Technologies). Using another conventional method for adipocyte differentiation, cells were exposed to adipogenic differentiation cocktails containing dexamethasone (1 *μ*M), IBMX (0.5 mM), insulin (5 *μ*g/ml) and 10% (v/v) FBS. The cells were maintained in reprogramming medium for 8 days beginning at day 0, and the media was exchanged every 48 h throughout all experiments (Figure 1a).

### Total RNA extraction and qRT-PCR analysis

Total RNAs from cells were extracted using TRIzol (Life Technologies) and a Direct-zol RNA MiniPrep Kit (Zymo Research, Irvine, CA, USA) with DNase I according to the manufacturer's recommendations. To perform the qRT-PCR assay, 400 ng of total RNAs was reverse-transcribed using the PrimeScript RT Reagent Kit and SYBR Premix Ex Taq (Takara Bio, Shiga, Japan) according to the manufacturer's recommendations. qRT-PCR was performed using a Thermal Cycler Dice Real Time System using the default cycling program (Takara Bio). The primers used in this experiment are listed in Supplementary Table 2. The relative gene expression levels of mouse total heart RNAs (Takara Bio) or human iPSC RNAs were normalized to *Gapdh* expression.

### Tissue preparation

Ten- to 12-week-old C57BL/6 mice were anesthetized and killed, and their hearts were removed at indicated time points. For total RNA and proteins extraction, the walls of the LV were dissociated from the whole heart. For total RNA isolation, the samples were cut into small pieces and homogenized with TRIzol using Bio Masher II (Nippi, Tokyo, Japan).

To isolate whole proteins, the samples were cut into small pieces and homogenized with lysis buffer (20 mM Tris-HCl (pH7.5), 137 mM NaCl, 10% glycerol (vol/vol), 1% NP-40 (vol/vol) (Wako Chemical Co.)), subsequently the lysates were sonicated with Bioruptor (CosmoBio Co. Ltd, Tokyo, Japan) for 4 min (30-s ON/30-s OFF) in ice-water.

For frozen sections, the kidneys were fixed with 4% paraformaldehyde (PFA; Wako Chemical Co.) for 2 h on ice, incubated overnight in 30% (vol/vol) sucrose in phosphate-buffered saline (PBS) at 4 °C and embedded in optimum cutting temperature compound (Sakura FineTek Japan Co., Ltd, Tokyo, Japan). Subsequently, 5-*μ*m thick sections were cut.

### Oil Red O staining and area calculation

Oil Red O powder (75 mg) was dissolved in 25 ml of 100% isopropyl alcohol and the solution was filtered to remove undissolved powder. PFA-fixed samples were washed with PBS and 60% (v/v) isopropyl alcohol. The samples were stained with 60% (v/v) Oil Red O solution for 15 min. Fat droplets in adipocytes were stained. Oil Red O-stained cells and frozen section samples were observed and images were captured with an IX71 inverted microscope (Olympus, Tokyo, Japan) or a BZ-X700 digital microscope (Keyence, Osaka, Japan). The percentage of total cell culture area positive for Oil Red O staining was calculated using ImageJ software (National Institutes of Health, Bethesda, MD, USA). At least three different wells were measured for each condition. For frozen sections, Mayer's hematoxylin was used as a counter-stain.

### Hierarchical clustering, PC and GO analyses

Gene expression analysis was performed using a SurePrint G3 Mouse GE Microarray Kit 8 × 60 K (Agilent Technologies, Santa Clara, CA, USA). Raw data were normalized and analyzed using GeneSpring GX11 software (Agilent Technologies). These normalized data were analyzed using the NIA (National Institute on Aging) Array Analysis website (http://lgsun. grc.nia.nih.gov/ANOVA/),^22^ a web-based tool for microarray data analysis using hierarchical clustering of averages and PCA. A hierarchical clustering analysis was performed using a minimum distance value of 0.001, a separation ratio of 0.5, and the standard definition of the correlation distance. GO and KEGG pathway enrichments were evaluated statistically following the instructions provided by the Database for Annotation, Visualization and Integrated Discovery (DAVID) 6.7.^57^ The gene expression microarray data have been submitted to the GEO (Gene Expression Omnibus) online database (http://www.ncbi.nlm.nih.gov/geo/) under accession number GSE70088.

### *In vitro* and *in vivo* IRI models

For the *in vitro* IRI model, CMPs were grown to 80% confluence and incubated in PBS for 3 h under hypoxic (1% O_2_, 5% CO_2_, balanced N_2_) conditions at 37 °C in a hypoxic chamber (ASTEC, Fukuoka, Japan), and subsequently incubated in growth media for 21 h under normoxic conditions (21% O_2_, 5% CO_2_). CMPs were collected at various time points (0, 3, 4, 6 and 24 h).^58^ Ten- to 12-week-old C57BL/6 mice were anesthetized by intraperitoneal injection of pentobarbital (50 mg/kg body weight) (Kyoritsu Seiyaku, Tokyo, Japan), and were intubated and ventilated under a respirator (SN-480-7, Shinano Manufacturing, Tokyo, Japan). General anesthesia was maintained by isoflurane. Following left thoracotomy, 7-0 Prolene suture thread was passed beneath the LAD just distal to the main trunk. The threads were tied transiently over a polyethylene tube for 60 min for ischemia, and were thereafter released for reperfusion. The LVs, including the areas at risk of IRI, were collected at various time points (0, 1, 1.5, 2 and 24 h) to examine gene profiles. At the end of the 24- h reperfusion period, Evans blue and TTC double staining was performed to verify IRI.^59^

### Western blotting

Samples (50 *μ*g) were mixed with bromophenol blue and 2-mercaptoethanol, boiled for 10 min, electrophoresed on 10% SDS polyacrylamide gel and electroblotted onto a PVDF transfer membrane (Millipore, Billerica, MA, USA). The membrane was blocked with PBS containing 5% skimmed milk, 0.05% Tween 20 and then incubated for 1 h with rabbit polyclonal antibodies to PPAR-*γ* (sc-7196; Santa Cruz Biotechnologies, Inc., Dallas, TX, USA), and mouse monoclonal antibodies to GAPDH (MAB374; Millipore), which were diluted to 1 : 500 with blocking buffer. After washing, the membrane was incubated with 1 : 5000 dilution of horseradish peroxidase (HRP)-conjugated donkey anti-rabbit IgG or HRP-conjugated donkey anti-mouse IgG (GE Healthcare, Little Chalfont, UK) in blocking buffer. Subsequently, the blots were developed using the ECL detection kit (GE Healthcare) and protein bands were visualized using the VersaDoc system (Bio-Rad Laboratories, Inc., Hercules, CA, USA).

### Statistical analysis

Results are expressed as mean values±S.E. The statistical significance of differences between groups was evaluated using Student's *t*-test, and *P*-values <0.05 were considered significant.

## Abbreviations

C/EBP: CCAAT/enhancer-binding protein
PPAR: peroxisome proliferator-activated receptor
Klf: Krüppel-like factor
KM: KLF4 and c-MYC
MSC: mesenchymal stem cell
TF: transcription factor
iPSC: induced pluripotent stem cell
OSKM: *Oct4, Sox2, Klf4* and *c-Myc*
CMP: cardiac mesenchymal progenitor
IRI: ischemic reperfusion injury
MI: myocardial infarction
qRT-PCR: quantitative reverse transcription polymerase chain reaction
PCA: principal component analysis
PC: principal component
GO: gene ontology
KEGG: Kyoto Encyclopedia of Genes and Genomes
LAD: left anterior descending artery
AON: area of necrosis
AAR: area at risk
RA: remote area
ROS: reactive oxygen species
LV: left ventricular
RAAS: renin–angiotensin–aldosterone system
NRX: nucleoredoxin
Dvl: dishevelled
bFGF: basic fibroblast growth factor
IBMX: 3-isobutyl-1-methylxanthine
DMEM: Dulbecco's modified Eagle's medium
FBS: fetal bovine serum
MEM: minimum essential media
KO-DMEM: knockout DMEM
PFA: paraformaldehyde
NIA array analysis: National Institute on Aging array analysis
DAVID: Database for Annotation, Visualization and Integrated Discovery
GEO: Gene Expression Omnibus
TTC: 2,3,5-triphenyltetrazolium chloride
