# Supplementary material for: Cardiac mesenchymal progenitors differentiate into adipocytes via Klf4 and c-Myc
Source: Cell Death Dis. 2016 Apr 14;7(4):e2190–. doi: 10.1038/cddis.2016.31 (PMC4855651; doi:10.1038/cddis.2016.31)
Supplement: Supplementary Figure 1 [file cddis201631x1.docx]

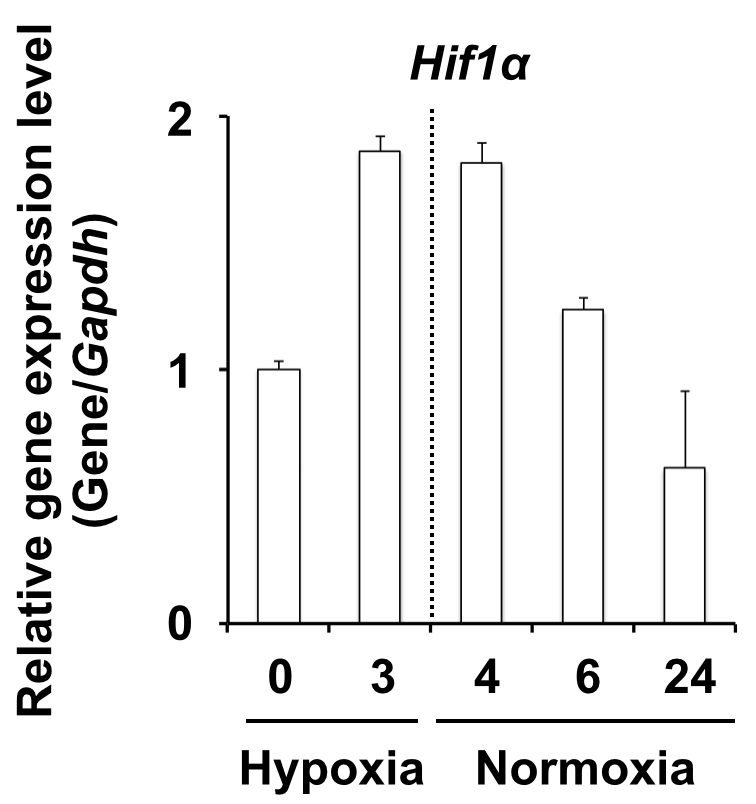


**Supplemental figure 1　Hypoxic gene expression of CMPs**

CMPs expressed the hypoxia-induced gene *Hif1α* at 3 h, and expression returned to baseline levels at 24 h.
