# Supplementary material for: Cardiac mesenchymal progenitors differentiate into adipocytes via Klf4 and c-Myc
Source: Cell Death Dis. 2016 Apr 14;7(4):e2190–. doi: 10.1038/cddis.2016.31 (PMC4855651; doi:10.1038/cddis.2016.31)
Supplement: Supplementary Figure 2 [file cddis201631x2.docx]

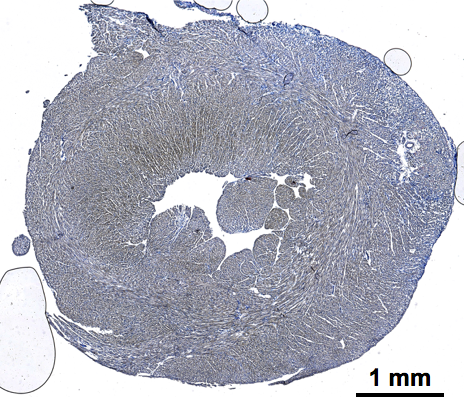


**Supplemental figure 2　Oil red O staining of murine hearts 2 days after LAD ligation**

Two days after LAD ligation, reperfused-hearts were not stained by Oil red O staining
