# Supplementary material for: Cardiac mesenchymal progenitors differentiate into adipocytes via Klf4 and c-Myc
Source: Cell Death Dis. 2016 Apr 14;7(4):e2190–. doi: 10.1038/cddis.2016.31 (PMC4855651; doi:10.1038/cddis.2016.31)
Supplement: Supplementary Table 2 [file cddis201631x3.docx]

**Supplemental table 2. Primer sets for quantitative RT-PCR**

| **Gene** | **Sequence (5’ to 3’)** |
| --- | --- |
| hOCT4-Forward | CTGGGTTGATCCTCGGACCT |
| hOCT4-Reverse | CACAGAACTCATACGGCGGG |
| hSOX2-Forward | CATGGACAGTTACGCGCACATGA |
| hSOX2-Reverse | TGGTAGTGCTGGGACATGTGAAGT |
| hc-MYC-Forward | AAGACTCCAGCGCCTTCTCTC |
| hc-MYC-Reverse | AGGAGCCTGCCTCTTTTCCAC |
| hKLF4-Forward | AATTACCCATCCTTCCTGCCC |
| hKLF4-Reverse | GTAATCACAAGTGTGGGTGGC |
| mC/Ebpα-Forward | TGGACAAGAACAGCAACGAG |
| mC/Ebpα-Reverse | AATCTCCTAGTCCTGGCTTG |
| mC/Ebpβ-Forward | CTTCAGCCCCTACCTGGAG |
| mC/Ebpβ-Reverse | GGAGAGGAAGTCGTGGTGC |
| mC/Ebpδ-Forward | CGACTTCAGCGCCTACATTGA |
| mC/Ebpδ-Reverse | CTAGCGACAGACCCCACAC |
| mFas-Forward | AAGGCTGGGCTCTATGGATT |
| mFas-Reverse | TGAGGCTGGGTTGATACCTC |
| mPparγ1-Forward | CTGCGTAACTGACAGCCTAAC |
| mPparγ1-Reverse | ACTTGGTCACTCTCCGTCCT |
| mPparγ2-Forward | CTGGGAGATTCTCCTGTTGACC |
| mPparγ2-Reverse | CCTTGCAGCAACATCAGGAA |
| mFabp4-Forward | CAGCCTTTCTCACCTGGAAG |
| mFabp4-Reverse | TTGTGGCAAAGCCCACTC |
| mMef2c-Forwad | TGTCCAGCCATAACAGTTTGG |
| mMef2c-Reverse | CCTTGTGAACATGAAGTCCTCTT |
| mTbx5-Forwad | ATGGCCGATACAGATGAGGG |
| mTbx5-Reverse | TTCGTGGAACTTCAGCCACAG |
| mGata4-Forwad | CACCCCAATCTCGATATGTTTGA |
| mGata4-Reverse | GGTTGATGCCGTTCATCTTGT |
| mHif1α-Forwad | ACCTTCATCGGAAACTCCAAAG |
| mHif1α-Reverse | CTGTTAGGCTGGGAAAAGTTAGG |
| c-Fos-Forwad | CGGGTTTCAACGCCGACTA |
| c-Fos-Reverse | TTGGCACTAGAGACGGACAGA |
| c-Jun-Forwad | CCTTCTACGACGATGCCCTC |
| c-Jun-Reverse | GGTTCAAGGTCATGCTCTGTTT |
| c-myc-Forwad | CAGAGGAGGAACGAGCTGAAGCGC |
| c-myc-Reverse | TTATGCACCAGAGTTTCGAAGCTGTTC |
|  |  |
|  |  |

h: Human, m: Mouse
